# Fracture resistance and fractographic analysis of pressable glass-ceramics with different partial coverage designs for maxillary premolars

**DOI:** 10.1186/s12903-024-04809-2

**Published:** 2024-09-13

**Authors:** Abdelaziz M. Abdelaal, Hany A. Kehela, Ahmed A. Holiel

**Affiliations:** Conservative Dentistry Department, Faculty of Dentistry, Alexandria University, Alexandria, Egypt

**Keywords:** Fracture resistance, Fractographic analysis, Lithium disilicate, Zirconia-reinforced lithium silicate, Partial coverage restorations

## Abstract

**Background:**

Partial coverage concepts have met the main goal of conservative dentistry. Vonlays, which combine features of veneers and onlays, are a recent alternative to full coverage designs and overlay partial coverage restorations. This in vitro study was conducted to compare the fracture resistance of the newly introduced pressable zirconia-reinforced lithium silicate with pressable lithium disilicate ceramic and to determine the optimal preparation design for partial coverage on upper premolars.

**Methods:**

Fifty-two duplicated epoxy resin dies were prepared following vonlay and overlay preparation guidelines. For each preparation (*n* = 26), the specimens were divided into two subgroups to be restored with lithium disilicate (IPS e.max Press) or zirconia-reinforced lithium silicate (Vita Ambria) (*n* = 13 each). Ceramic vonlays and overlays were bonded using dual cure resin cement, subjected to thermomechanical fatigue, and the load to fracture was tested by using a universal testing machine. The specimens were fractographically analyzed via scanning electron microscopy (SEM). The normality of the fracture resistance data was checked using the Shapiro‒Wilk test and Q‒Q plots, and two-way ANOVA was used to assess the effect of the type of preparation and ceramic material on the fracture resistance.

**Results:**

The group of overlays restored with zirconia-reinforced lithium silicate showed the highest mean fracture load (1218.69 N), while the group of vonlays restored with lithium disilicate had the lowest mean fracture resistance (967.15 N). The effect of preparation design and material type on fracture resistance was significant for both factors, *p* = 0.003 and *p* < 0.0001, respectively. Different features of the fracture surfaces, such as arrest lines, hackles, and directions of crack propagation, were observed.

**Conclusions:**

Zirconia-reinforced lithium silicate exhibited greater resistance to fracture compared to lithium disilicate, making it a potential substitute for partial coverage restorations. Additionally, the overlay showed superior fracture resistance when compared to the vonlay preparation design.

## Introduction

Partial coverage preparation designs restored with dental ceramics may be considered a breakthrough in recent dental practice, meeting the main goal of the novel trend of minimally invasive dentistry [1]. There is a well-established link between increased tooth structure loss and strength degradation [2]. Furthermore, cavity preparation considerably reduces cusp stiffness [3, 4]. Therefore, expanding preparation designs from inlays and partial coverage onlays to complete-coverage crown restorations, traditionally used to strengthen the tooth/restoration complex, often comes at the expense of the remaining tooth structure [5]. This approach frequently leads to fracture failures that affect both the restoration and the underlying tooth structure, typically resulting in catastrophic outcomes [6]. Furthermore, wide complete coverage crown preparation designs may compromise the health of the tooth [7].

The term “Vonlay,” also referred to as “Veneerlay,” represents a recently introduced partial coverage preparation design, specifically tailored for the posterior region. It involves combining an onlay with an extended buccal veneer surface, suitable for areas where there is sufficient enamel for bonding [8]. Although this restorative approach offers the similar esthetics and structural benefits as a full coverage crown, it involves a far less intrusive preparation. The components from veneers and onlays are combined to improve the preserved tooth structure’s appearance while providing function and longevity [9, 10]. Moreover, various contemporary overlay preparation designs have been introduced in several studies as acceptable partial restorations in the posterior region with improved mechanical behavior [11, 12].

Although various types of dental ceramic materials have been introduced in recent years being indicated for different types of indirect restorations, lithium disilicate glass ceramics have achieved appropriate agreement due to a relevant fracture resistance [13]. This may be attributed to the highly filled glass matrix, made possible by the shape and volume of crystals, resulting in a crystalline content of approximately 70% [14]. Clinical and scientific data available for different restorations fabricated with this material are considered excellent [15]. Recently, zirconia-reinforced lithium silicate glass ceramic materials (ZLS) were introduced, where 10% zirconium dioxide particles are homogeneously incorporated into a lithium metasilicate glass ceramic matrix to improve its biocompatibility and mechanical properties [16, 17]. Several studies have been conducted to evaluate the fracture resistance of CAD/CAM ZLS restorations [18, 19], but none has experienced the fracture resistance of the pressable ZLS.

The curse of brittle ceramic dental materials is that they are prone to catastrophic fracture. Fracture can be defined as the detrimental process of creating new surfaces within a body [20]. Fracture usually occurs at stresses above the elastic limit of materials, where the stress at which a material fractures is referred to as the fracture strength (resistance) [21]. The fracture surfaces of broken parts can be examined by fractographic analysis because much information can be obtained through observation and quantification of the extension, direction, and patterns of crack propagation within fractured surfaces [20].

The purpose of this in vitro study was to compare the fracture resistance of two types of pressable ceramics, zirconia-reinforced lithium silicate and lithium disilicate and to determine the optimal preparation design for partial coverage (vonlays and overlays) restoring maxillary premolars, as well as to evaluate their fractographic behavior. The null hypothesis states that there is no difference in the fracture resistance of the two ceramic materials used for vonlays and overlays.

## Methods

### Teeth selection and preparation

Two maxillary premolars, extracted for orthodontic reasons, were collected from informed patients who agreed to voluntarily donate their teeth for research purposes, respecting the Declaration of Helsinki, with explicit consent obtained for their utilization. The teeth were inspected and checked to be free of caries, cracks, and restorations. The teeth were kept hydrated during the experimental procedures by immersion in saline solution [22].

**Fig. 1:**
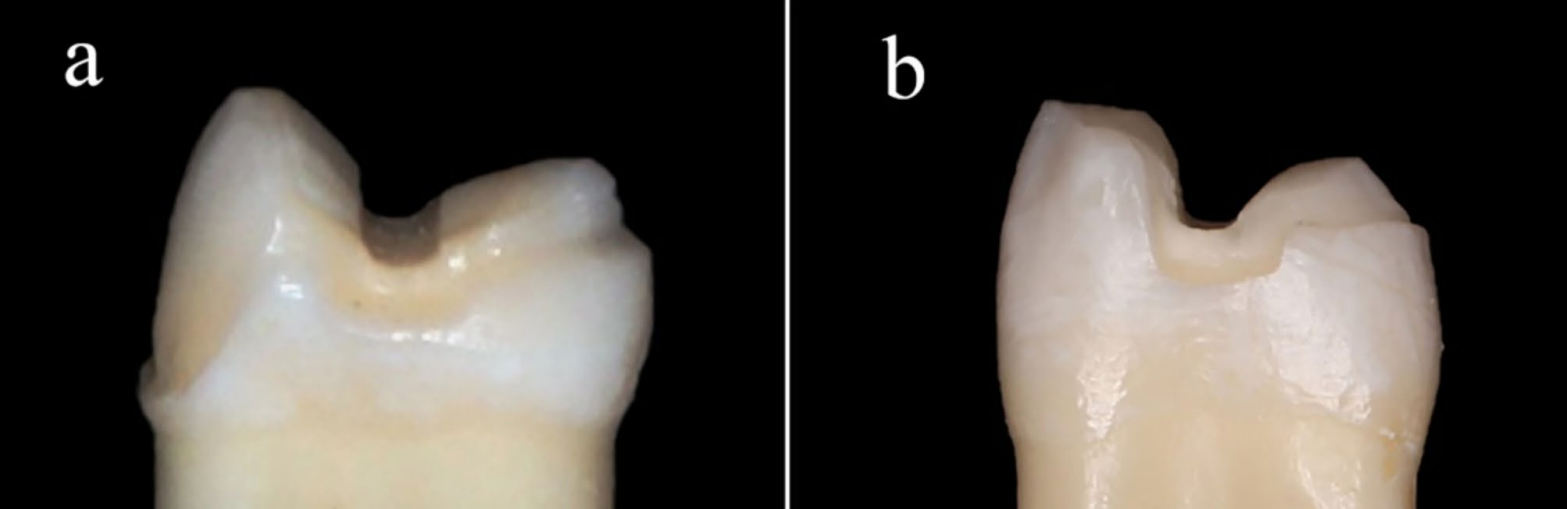
(**a**) a vonlay tooth preparation, (**b**) overlay tooth preparation

Teeth were prepared as a vonlay and an overlay preparation design. Vonlay preparation **(**Fig. 1a**)** was performed following the ceramic MOD inlay guidelines with buccal and palatal cuspal reduction following occlusal anatomy [9]. The reduction dimensions were 1.5 mm for the buccal (nonfunctional) cusp and 2 mm for the palatal (functional) cusp using a tapered flat end diamond bur (ISO 171/016, TF-21, Mani, Germany). The depth of the occlusal box was 2 mm from the cusp tip to the pulpal floor, mesial and distal boxes measured 1 mm from the pulpal floor to the gingival seat. The divergence angle (12˚) was made using a flat-end conical diamond bur (ISO 171/016, TF-31, Mani, Germany), where the isthmus portions extended for one third of the bucco-palatal dimension. The preparation was extended to the labial surface to end with a chamfer finish line of depth 0.5 mm using a tapered round-end diamond bur (ISO 199/016, TR-12, Mani, Germany) 1 mm away from the cementoenamel junction in the occlusal direction. The finish line on the palatal cusp was chamfer located 1 mm from the occlusal contact on the functional cusp and was extended to meet the mesial and distal boxes. All the measurements were made using a calibrated periodontal probe, and the internal line angles and the margins of the preparation were rounded and finished [9]. The Overlay preparation **(**Fig. 1b**)** was prepared with the same mentioned preparation guidelines with a chamfer finish line located 1 mm from the occlusal contact on the functional cusp, which was extended to meet the mesial and distal boxes and a reduction of 1.5 mm to the buccal cusp without a finish line [11]. The preparations were performed by the same operator.

### Duplication of the prepared teeth

Teeth were duplicated to form twenty-six epoxy resin dies for each preparation design. Half of the specimens were restored with lithium disilicate, and the other were restored with zirconia-reinforced lithium silicate. The sample size was calculated based on a previous study that compared the fracture resistance of ceramic vonlays restoring premolars [9]. Sample size was based on Rosner’s method [23] calculated by G*Power 3.1.9.7. [24] By assuming 5% alpha error and 80% study power, the minimal required sample size was found to be 11 samples per group, which was increased to 13 samples to compensate for processing errors. Total sample = number per group × number of groups = 4 × 13 = 52 samples.

Polyvinyl siloxane (PVS) molds (Coltene/Whaledent AG, 9450 Altstatten, Switzerland) were used for the construction of the epoxy resin dies. After mixing, the material was injected into a cylindrical plastic container with a diameter of 20 mm, and the prepared natural tooth was placed. The material was allowed to set, after which the natural tooth was removed. This procedure was repeated to produce 26 silicon molds for each preparation design [9]. The base and the catalyst of the epoxy resin material (solvent-free transparent epoxy resin. CMB Intl, Egypt) were mixed in an auto mixing device (200 r/min) and then poured into the PVS molds under vibration to eliminate air bubbles. The epoxy resin dies were allowed to completely set for 48 h [9]. Magnifying loupes (3×) were used to check for any surface defects in the epoxy resin dies, after which epoxy resin bases were constructed to support the dies during the cyclic loading and fracture resistance tests. A plastic cylinder was used as a holder for the epoxy resin material, forming the base, with the epoxy resin die placed at its center until the epoxy resin was completely set [9].

### Grouping

A total of 52 epoxy resin dies were divided into four groups according to the preparation design and the type of restorative material used: Evon- Vonlay cavity preparation design restored with lithium disilicate (IPS e.max Press, Ivoclar Vivadent, Schaan, Liechtenstein); VVon- Vonlay cavity preparation design restored with zirconia-reinforced lithium silicate (Vita Ambria, VITA Zahnfabrik, Bad Säckingen, Germany); EOver- Overlay cavity preparation design restored with lithium disilicate (IPS e.max Press); VOver- Overlay cavity preparation design restored with zirconia-reinforced lithium silicate (Vita Ambria).

### Optical impression of the epoxy resin dies and designing of wax

**Fig. 2:**
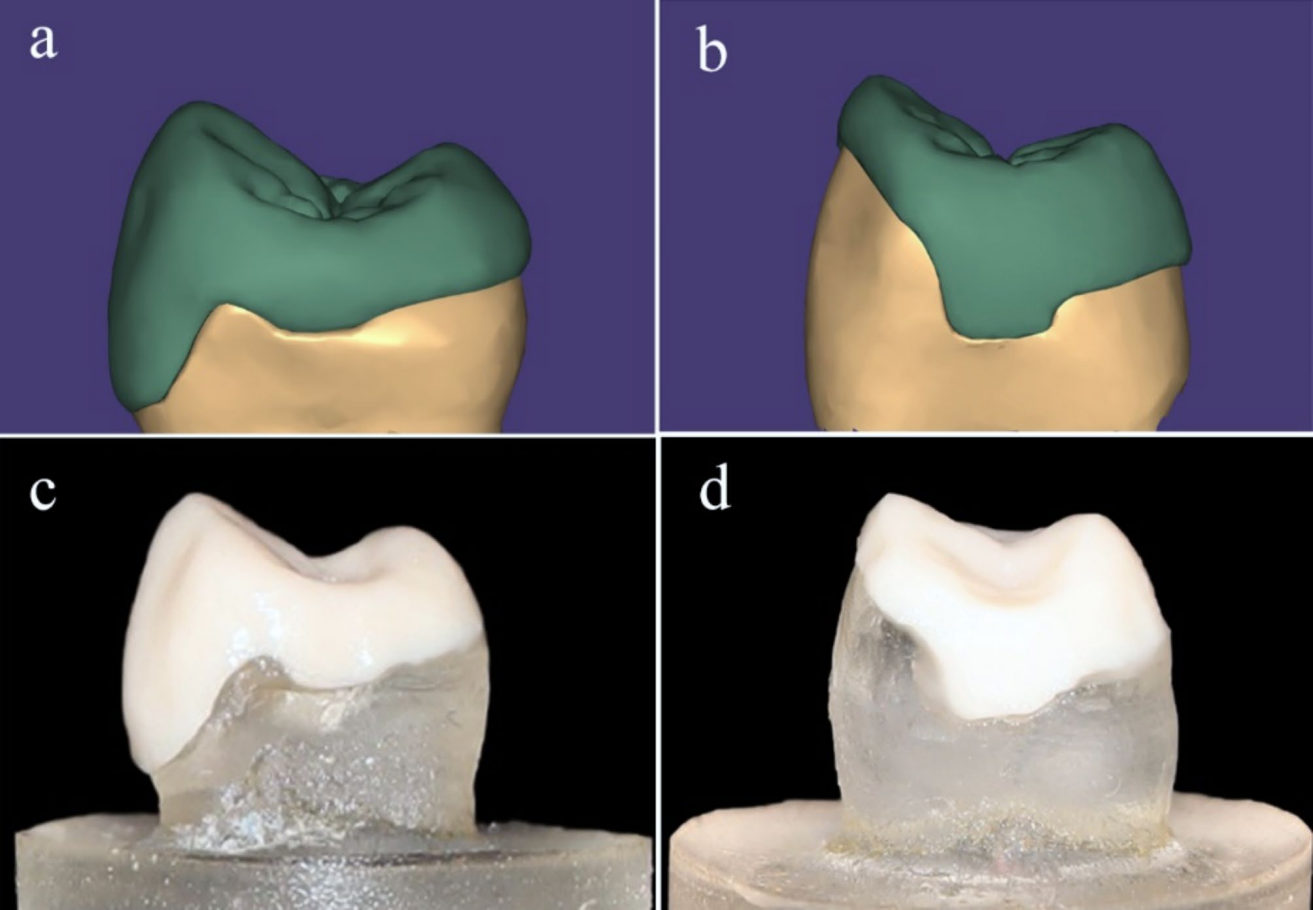
(**a**, **b**) showing virtual wax pattern designs for vonlay and overlay respectively, (**c**, **d**) cemented vonlay and overlay

Digital impressions were made to construct wax patterns for the fabrication of the ceramic restorations [9]. Each die was sprayed with Cerec Optispray (Sirona dental systems GmBH, Germany) and scanned using an intraoral scanner (CarestreamCS3700, Carestream Dental LLC, Atlanta, GA, USA). The scanning procedure was continuous starting from the occlusal surface of the die with alternating bucco-palatal movement followed by the proximal surfaces of each die [25]. The wax patterns were digitally designed with computer-aided design software (Fig. 2a and b). The designs were dry milled in CAD/CAM wax blanks (Super Green wax, Natura DMAX, Daegu, Korea) to produce 26 wax specimens for each preparation design using a 5-axis milling machine (Ceramill Motion 2 - Amann Girrbach AG, Austria) [26].

### Fabrication of the ceramic vonlays and overlays

Wax patterns of the vonlays and overlays were invested (Bellavest T, BEGO, Germany) and burned out. Lithium disilicate ingots were pressed and the restorations were constructed using firing and pressing furnace (Programat EP 3010; Ivoclar Vivadent, Schaan, Liechtenstein) at a temperature of 850˚C for 60 min according to the manufacturer’s instructions. Additionally, ZLS ingots were pressed, and the restorations were constructed at a temperature of 880˚C for 25 min in the same furnace. After divestment and separation, the restorations were prepared for cementation, after which the fitting surfaces of the restorations were etched with 9.5% hydrofluoric acid (Bisco, Schaumburg, Illinois, USA) for 90 s, thoroughly rinsed with water, and air-dried. The etched intaglio surfaces appeared dull and frosty, then a silane coupling porcelain primer (Bisco, Schaumburg, Illinois, USA) was applied combined with one layer of porcelain bonding resin (Bisco, Schaumburg, Illinois, USA) [27]. Ceramic vonlays and overlays were bonded using dual cure resin cement (BisCem, Bisco, Schaumburg, Illinois, USA) (Fig. 2c and d) with a specially designed device for load application during the cementation procedure.

### Thermo-mechanical fatigue and fracture resistance test

The specimens were subjected to 250,000 mechanical cycles at 1.6 Hz frequency, and simultaneous 1000 thermal cycles between 5 °C and 55 °C with 60 s holding time and 15 s between temperatures in a customized mastication simulator that simulates approximately 12 months of clinical conditions [28]. A stainless steel antagonist represented the opposing cuspwas directed to make initial contact with the internal incline of the palatal (functional) cusp [29]. A 0.5 mm thick silicon sheet was placed between the loading point and the surface of the specimens to prevent generation of contact surface damage which could lead to premature failure [30].

All specimens were visually inspected under a light stereomicroscope (SZ1145TR, Olympus Japan) at 5–10× magnification to detect any cracks or restoration debonding to be excluded from the fracture resistance testing procedure. All the specimens underwent load-to-fracture testing under a universal testing machine (5 ST, Tinius Olsen England 2018). A stainless steel sphere with a diameter of 4 mm in the upper compartment of the device was used to apply the compressive load axially at a crosshead speed of 1 mm/min, making contact with the center of the occlusal surface of each specimen [31]. During the testing procedure, the load gradually increased until a sudden sharp drop in the force was recorded within the software, and this change was accompanied by failure. The maximum load (N) recorded before the reduction in the magnitude of the force was considered the “fracture load” [12].

### Fractographic analysis

After failure, each specimen was inspected under a stereomicroscope (18×), then coated with gold and inspected under a scanning electron microscope (SEM, JEOL-JSM-5510LV, JEOL Ltd., Tokyo, Japan) (35× and 500× magnifications). The mode of failure of each specimen was categorized following Burke’s classification [32] as follows: Type I- minimal fracture or crack in the crown; Type II- less than half of the crown lost; Type III- crown fracture through midline or half of the crown displaced or lost; Type IV- more than half of the crown lost; Type V- severe fracture of the crown and/or tooth. In addition, cracks, chipping, delamination, and catastrophic total failures were noted.

### Statistical analysis

The normality of the fracture resistance data was checked using the Shapiro-Wilk test and Q‒Q plots and was found to be normally distributed. The data are presented mainly as the mean, standard deviation and 95% confidence interval (CI). The mode of fracture was presented as the frequency and percentage. Two-way analysis of variance (ANOVA) was used to assess the effect of the type of preparation and restorative materials on the fracture resistance. The Kruskal-Wallis test with Dunn’s post hoc test with Bonferroni correction was used to analyze the failure mode between groups. All tests were two tailed, and the level of significance was set at a *p* value ≤ 0.05. The data were analyzed using IBM SPSS, version 23 for Windows (Armonk, NY, USA).

## Results

A comparison of fracture resistance among the four groups is presented in Table 1, which shows the mean (N) and standard deviation for each group according to the 95% confidence intervals, medians, and minimum and maximum values. The VOver group had the highest mean value (1218.69 ± 87.30 N), followed by the VVon group which was (1123.08 ± 142.73 N) and the EOver group (1033.38 ± 83.13 N); the lowest mean value was recorded for the EVon group (967.15 ± 33.14 N).

**Table 1:** Comparison of fracture resistance (N) among the study groups

	Vonlay		Overlay	
	E-max (*n*** = 13**)	Vita Ambria (*n*** = 13**)	E-max (*n*** = 13**)	Vita Ambria (*n*** = 13**)
Mean	967.15 ± 33.14	1123.08 ± 142.73	1033.38 ± 83.13	1218.69 ± 87.30
95% CI	947.13, 987.18	1036.82, 1209.33	983.15, 1083.62	1165.94, 1271.45
Median	961.00	1101.00	997.00	1232.00
Min – Max	925.00–1048.00	941.00–1345.00	901.00–1170.00	1013.00–1332.00

Two-way ANOVA is shown in Table 2 to evaluate the effect of preparation design and material type on fracture resistance, which was statistically significant for both F test = 9.46 (*p* = 0.003) and F test = 42.04 (*p* < 0.0001), respectively, where Table 3 shows the estimated marginal means of preparation and material. The partial eta squared value is 0.165 for the preparation design and 0.467 for the material type. The interaction effect between these two variables was not significant (*p* = 0.579) according to the partial eta squared (0.006) test.

**Table 2:** Two-way ANOVA assessing the effect of preparation and material on fracture resistance

Variables	Mean square	Df	F test	*P* value	Partial Eta Squared
Vonlay vs. Overlay	85131.08	1	9.46	0.003*	0.165
E-max vs. Ambria	378424.92	1	42.04	< 0.0001*	0.467
Interaction	2806.23	1	0.312	0.579	0.006
Corrected Model	155454.08	3	17.27	< 0.0001*	0.519

*Statistically significant difference at *p* value ≤ 0.05

**Table 3:** Estimated marginal means of preparation and material

Variables		Mean	SE	95% CI
Preparation	Vonlay	1045.12	18.61	1007.71, 1082.53
	Overlay	1126.04	18.61	1088.63, 1163.45
	*Difference*	80.92	26.31	28.02, 133.83
Material	E-max	1000.27	18.61	962.86, 1037.68
	Ambria	1170.89	18.61	1133.48, 1208.29
	*Difference*	170.62	26.31	117.71, 223.52

**Fig. 3:**
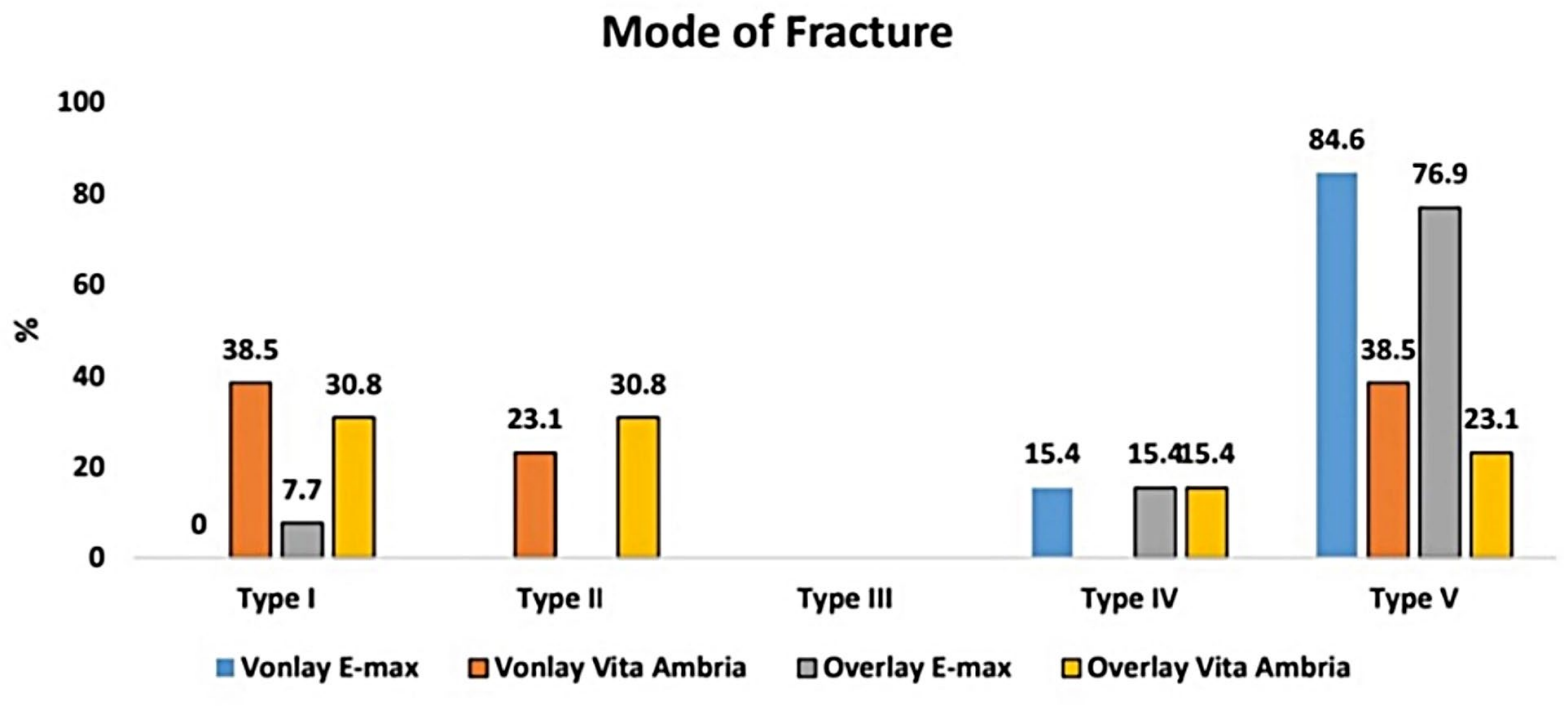
A bar chart of each Burke’s classification score, with color-coded bars representing its percentage in each of the study groups

**Fig. 4:**
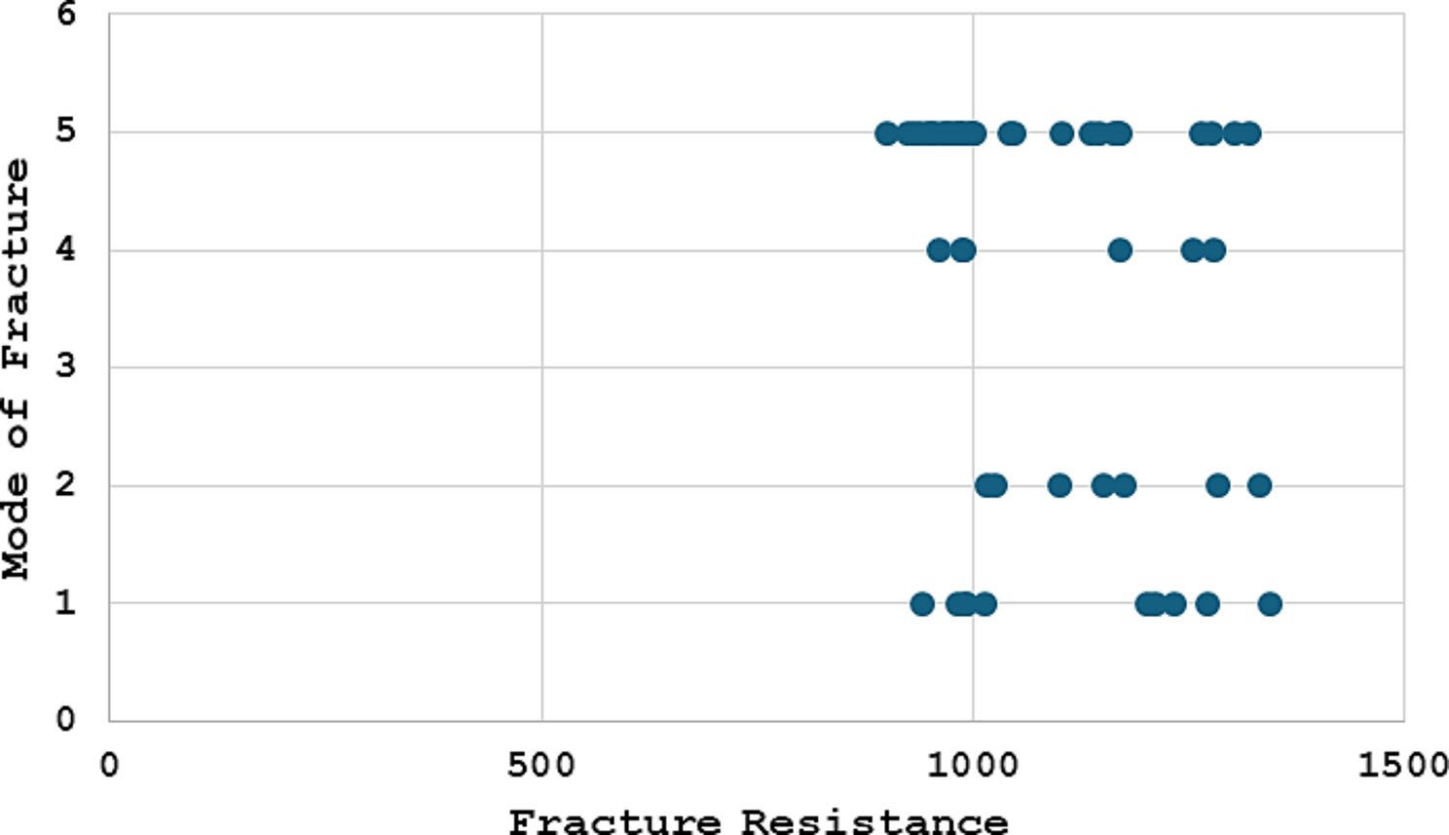
Correlation between mode of fracture and fracture resistance

A comparison of the mode of fracture between the four study groups is presented in Fig. 3, which shows the number of samples located at each type of Burke’s classification and its percentage among the groups; these results are statistically significant (*p* = 0.001). A pairwise comparison of the mode of fracture according to Burke’s classification among the study groups showed that the difference between EVon and VVon was significant (*p* = 0.026); however, when compared with EOver, the difference was not significant (*p* = 1.00). The difference between the EVon and VOver groups was significant (*p* = 0.007). A comparison between the VVon and the EOver and VOver groups did not reveal significant differences (*p* = 0.100 and *p* = 1.00, respectively), while a significant difference was found when comparing the EOver to the VOver group (*p* = 0.033). Additionally, a negative correlation between fracture resistance and failure mode was identified, with a Rho value of -0.306 (*p* = 0.027), as shown in Fig. 4.

**Fig. 5:**
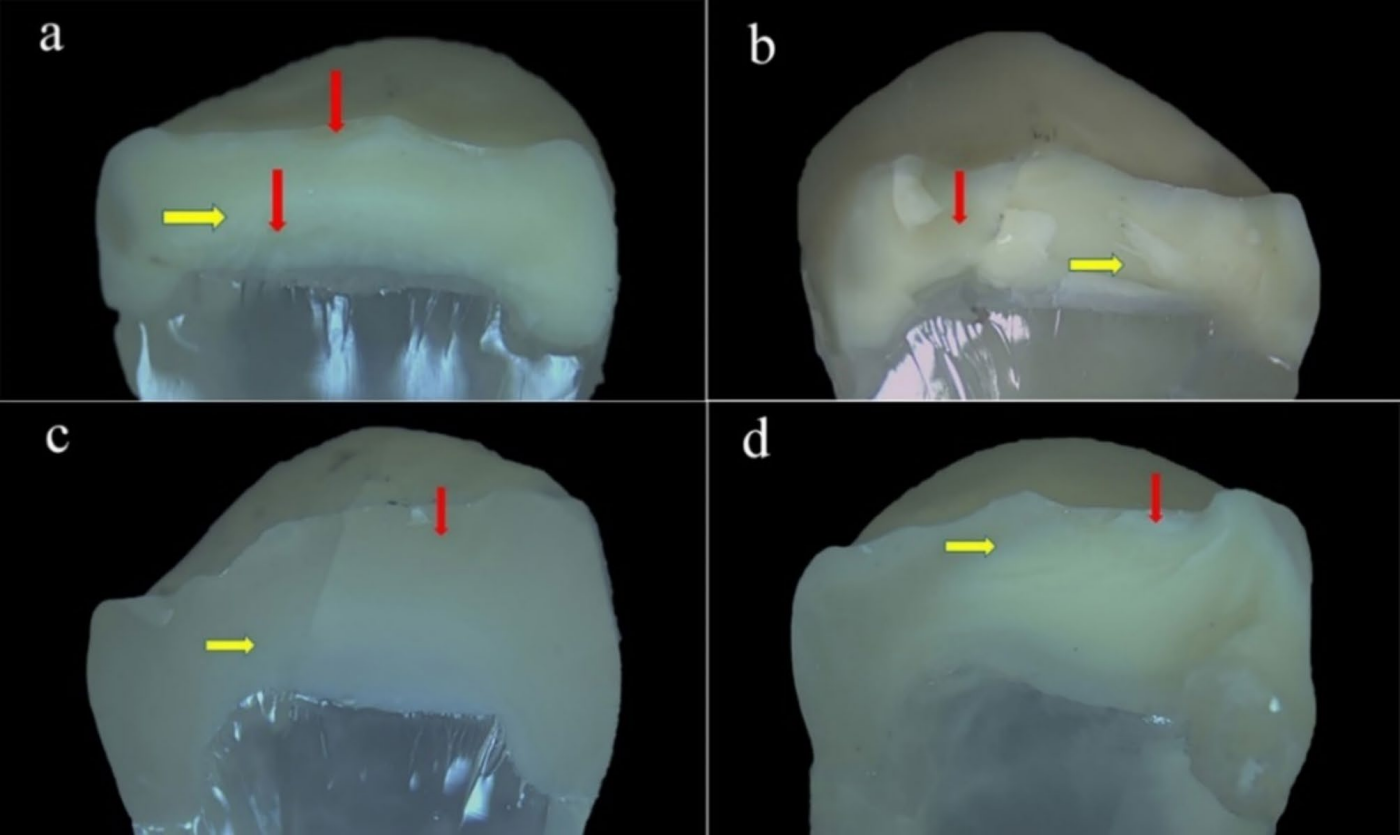
Stereomicroscope images (18×) of representative samples from each group. (**a**) for EVon group, (**b**) for VVon group, (**c**) for EOver group, (**d**) for VOver group. Red arrows: Arrest lines; yellow arrows: Hackle lines

**Fig. 6:**
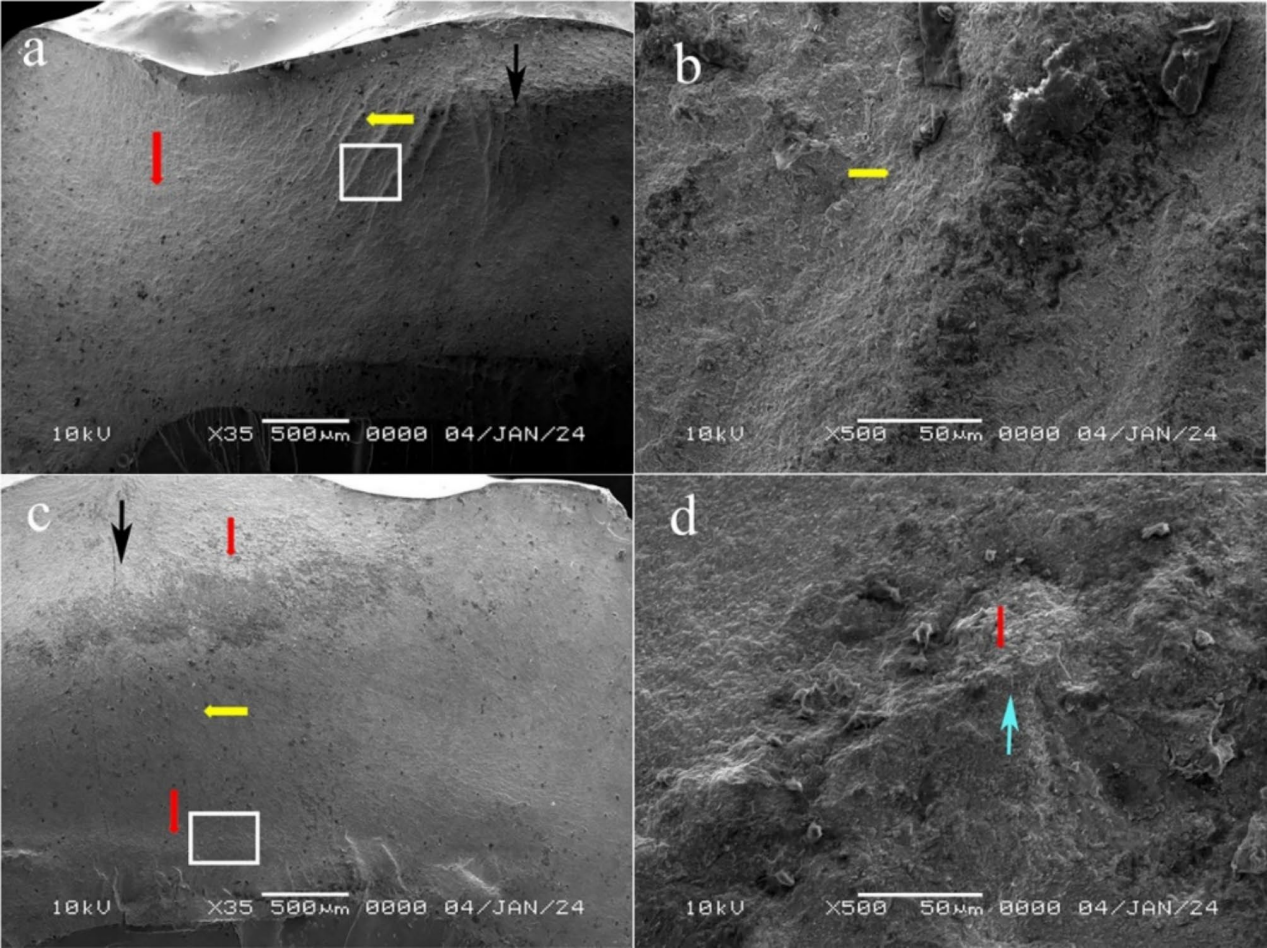
SEM images of representative samples from groups EVon and VVon at 35× magnification (Fig. 6 a and c, respectively), where the white squared area is shown at 500× magnification (Fig. 6 b and d, respectively). Red arrows: arrest lines; yellow arrows: hackle; black arrow: direction of crack propagation, blue arrow: twist hackle

**Fig. 7:**
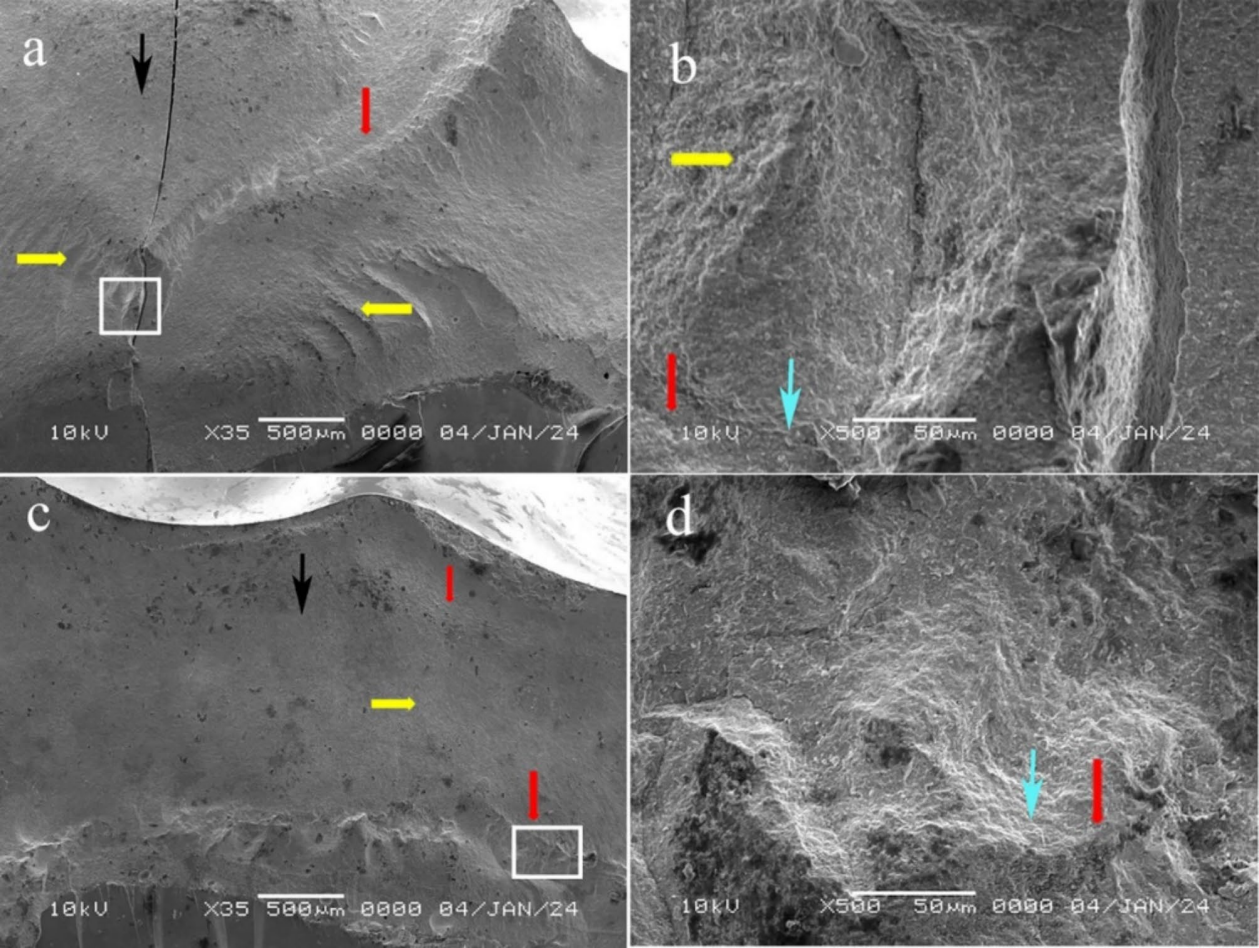
SEM images of representative samples from groups EOver and VOver at 35× magnification (Fig. 7 a and c, respectively), where the white squared area is shown at 500× magnification (Fig. 7 b and d, respectively). Red arrows: arrest lines; yellow arrows: hackle; black arrow: direction of crack propagation, blue arrow: twist hackle

Fractographic analysis revealed the detailed features of the fractured surfaces and indicated that the fracture originated at the occlusal side (at the loading sphere placement area) and propagated cervically. Different fracture features, such as arrest lines (Figs. 5 and 6a, c and d **and** Fig. 7), hackles (Figs. 5 and 6a, b and c **and** Fig. 7a, b and c) and twist hackles, were indentified at higher magnifications (Fig. 6d **and** Fig. 7b and d). Chipping was not observed in the specimens, while cracks were observed in the VVon (3 specimens) and VOver (3 specimens) groups and were classified as type I according to Burke’s classification. Fracture of the restoration without displacement (also classified as type I according to Burke’s classification) was observed in the VVon (2 specimens), EOver (1 specimen) and VOver (1 specimen) groups, where the fracture was complete along the depth of the restoration mesiodistally. **Type II fractures** were found in the VVon and VOver groups, where less than half of the restoration fractured and displaced, with the rest remaining attached. **Type III failures** were absent in all groups. **Type IV failures** occurred in the EVon, EOver, and VOver groups, with varying degrees of restoration displacement and fragmentation. **Type V catastrophic failures** were present in all groups, with the EVon group showing the most significant damage, followed by EOver, VVon, and VOver groups, where both the restorations and epoxy resin dies experienced complete fractures, often involving displacement.

## Discussion

The aim of this study was to compare the fracture resistance of Vita Ambria, a newly introduced material, to IPS e.max Press, often regarded as the gold standard for glass-ceramic pressable materials. Additionally, the study evaluated the fracture resistance of two partial coverage preparation designs, vonlays and overlays, used for restoring premolars. Various factors can influence the fracture behavior of ceramic restorations, including the choice of restoration and die materials, preparation geometry, restoration thickness, and cementation procedures [10]. The natural tooth material significantly affects stress distribution in restorations, making it essential to consider this when simulating in vivo conditions. In this study, an epoxy resin die with a lower elasticity modulus was used to replicate clinical conditions, ensuring standardization across samples and facilitating reproducibility [33]. A single virtual restoration design for each preparation design was created using CAD/CAM system and milled from a wax blank to standardize the ceramic thickness for all samples, which were then pressed using ceramic ingots.

This study indicated that Vita Ambria exhibited significantly greater fracture resistance compared to E-max Press for both preparation designs. Additionally, the overlay preparation design demonstrated significantly higher fracture resistance than the vonlay preparation design. Therefore, the null hypothesis proposed was rejected. This could be attributed to the incorporation of zirconia particles in the Vita Ambria ceramic, which could hinder the crack propagation process, requiring higher load values to failure [34]. Zirconia particles act as nucleating agents incorporated in the glassy matrix causing crack propagation interruption [35]. Zirconium dioxide (ZrO_2_) transformation toughening reinforces the glass-ceramic in addition to the action of crack deflection. Compressive stress is created on the crack or in the micro cracks surrounding it by the expansion of the ZrO2 grain volume that accompanies the phase transition. These micro cracks increase the glass-ceramic’s resistance to fracture by absorbing the energy from the main crack [36, 37].

The findings of this study align with a previous investigation [16], which concluded that Vita Suprinity showed significantly greater fracture resistance than IPS e.max CAD. The authors attributed this finding to the fine homogeneous crystalline structure of the former in comparison to the needle-shaped crystals embedded in the glassy matrix of the latter. Additionally, the high fracture toughness of ZLS is related to the incorporation of zirconia particles, which reinforce the glassy matrix without being clouded by the dissolution of the zirconia particles [17]. Another study [18], also reported that ZLS crowns exhibited greater fracture resistance than those made of lithium disilicate and justified their results due to the variation in the microstructure of both materials. A study by Ghajghouj and Taşar-Faruk [19], showed greater fracture resistance for ZLS endocrowns compared to lithium disilicate endocrowns. Another study conducted by Al-Akhali et al. [14], inferred that lithium disilicate had greater fracture resistance than ZLS before thermomechanical fatigue; however, lower fracture resistance was verified for lithium disilicate after thermomechanical aging.

All preparation designs in our study followed standardized dimensions and included an MOD inlay box to simulate complex clinical scenarios and assess the performance of various ceramic materials under challenging conditions. The overlay preparation design has shown greater fracture resistance than the vonlay preparation design. This result contrasts with the findings of a previous study [10], which reported that occlusal onlays (overlays) had lower failure loads than complete veneers (vonlays) when both were restored with IPS e.max Press using standard preparation dimensions. However, in cases of minimal thickness, overlays exhibited better performance than vonlays. The results of our study may be attributed to the complexity of the vonlay preparation and the reduced thickness of the buccal portion, as well as the delicacy of the transitional parts between the occlusal onlay portion and the veneer portion, which may lead to catastrophic failures. Specifically, the preparations’ details could create stress concentrators, particularly if the transitions are not smoothly executed or if a more brittle material is used. This explanation is consistent with the findings of a previous study [12], which analyzed the fracture behavior of occlusal veneers with different designs and concluded that an increase in the number of axial walls in the preparation design led to a decrease in the fracture resistance with more fracture stresses within the restoration.

Fractographic analysis, typically used to determine crack propagation direction and fracture origin, was conducted in this study using SEM on the fracture surfaces of pressable glass-ceramics. The fractures were found to originate at the occlusal side, where the loading sphere was placed, and then propagated cervically. Key features observed included smooth mirror regions indicating initial crack growth, mist regions with microcracks, and hackle regions with secondary cracks. However, the high crystalline content of glass ceramics made detailed fractographic analysis challenging with SEM imaging [20]. The study revealed that fracture modes varied based on preparation design and restorative material. ZLS showed fractures originating from stress concentration points with higher resistance to crack propagation and less branching, indicating a more favorable failure mode. In contrast, conventional lithium disilicate fractures often propagated through larger areas, starting from surface defects or stress concentrations, reflecting its lower fracture resistance compared to ZLS. This suggests a negative correlation between fracture resistance and failure mode, where increased fracture resistance correlates with less severe failure modes. The study also highlighted the impact of preparation design on material performance, with smoother designs like overlay offering better stress distribution and reduced failure severity. While vonlays frequently showed fractures starting at the transition between the buccal surface and the proximal box, with cracks following stress concentration lines. This pattern supports the design’s lower fracture resistance and more severe failure modes. These findings align with previous research [12], which experimented with different preparation designs of maxillary premolars and found that the fracture occurred directly beneath the loading area and also in accordance with Kasem et al. [38], who evaluated the fracture resistance of a ZLS material (celtra duo) and found that fractures originated mainly at the occlusal side at the area where the indenter was placed and propagated towards the cervical line.

The way each specimen has experienced failure (Burke’s classification) may not closely simulate the in vivo situation; however, it is a standardized method to compare various groups [32]. The absence of Type III failures in this study is likely due to material properties, experimental conditions, and the fracture behavior of the restorations. This finding aligns with a previous study [9], which also found no Type III failures in ZLS VITA Suprinity and IPS e.max CAD vonlays. If applicable on clinical situation, types I, II and IV represent fracture of the restoration without fracture of the underlying prepared tooth, which could be clinically treated by repairing or replacing the restoration, while type V include the fracture of the prepared tooth which may be untreatable.

A limitation of this study, as with most in vitro studies, is the inability to completely simulate the oral environment even performing thermomechanical aging. However, in vitro studies are considered a reliable method for comparing different groups and provide an indication of the material’s mechanical behavior under certain conditions. Another limitation was the use of epoxy resin dies instead of natural teeth, which, despite ensuring standardization, may have caused some variation from the clinical response due to the specific characteristics of bonding to dental tissues and their behavior after thermomechanical fatigue. Further studies could integrate finite element analysis (FEA) to correlate the experimental results with computational models, offering a more thorough understanding of the mechanical behavior of the restorations. This would extend the findings of the current research. Additionally, future research should include clinically relevant scenarios, such as using teeth with carious lesions or other forms of damage, to better replicate clinical conditions. This would provide more detailed information on how disease impacts preparation, bonding, and restorative material performance. Moreover, further investigation is required to assess the performance of other commercially available products, enabling a comparative analysis of different brands and providing a deeper insight into how material variations impact clinical outcomes.

## Conclusions

Zirconia-reinforced lithium silicate exhibited greater resistance to fracture compared to conventional pressable lithium disilicate, making it a potential substitute for partial coverage restorations. Additionally, the overlay preparation design showed superior fracture resistance compared to the vonlay preparation design. Future research incorporating diverse tooth models, multidirectional loading, and natural teeth is needed to validate and extend these findings for broader clinical applicability.

## Data Availability

All the data generated or analyzed during this study are included in this article. Further inquiries can be directed to the corresponding author.

## Abbreviations

ZLS: Zirconia-reinforced lithium silicate glass ceramic materials
Group EVon: Vonlay cavity preparation design restored with lithium disilicate (IPS e.max Press)
Group VVon: Vonlay cavity preparation design restored with zirconia-reinforced lithium silicate
Group EOver: Overlay cavity preparation design restored with lithium disilicate (IPS e.max Press)
Group VOver: Overlay cavity preparation design restored with zirconia-reinforced lithium silicate
